# Amplitude modulated gamma oscillations as electrophysiological markers for repetitive transcranial magnetic stimulation efficacy in treatment-resistant depression: a randomized sham-controlled study

**DOI:** 10.1016/j.ijchp.2025.100593

**Published:** 2025-06-16

**Authors:** Yi-Chun Tsai, Cheng-Ta Li, Wei-Kuang Liang, Chih-Ming Cheng, Jia-Shyun Jeng, Chi-Hung Juan

**Affiliations:** a Cognitive Intelligence and Precision Healthcare Center, National Central University, Taoyuan City 32001, Taiwan; b Department of Psychiatry, Taipei Veterans General Hospital, Taipei, Taiwan; c Institute of Cognitive Neuroscience, College of Health Sciences and Technology, National Central University_,_ Taoyuan City 32001, Taiwan; d Division of Psychiatry, Faculty of Medicine, National Yang-Ming Chiao-Tung University, Taipei, Taiwan; e Department of Psychiatry, College of Medicine, National Yang-Ming Chiao-Tung University, Taipei, Taiwan; f Department of Psychology, Kaohsiung Medical University, Taiwan

**Keywords:** Amplitude modulation (AM), Gamma oscillations, Holo–Hilbert spectral analysis (HHSA), Repetitive transcranial magnetic stimulation (rTMS), Treatment-resistant depression (TRD)

## Abstract

**Background:**

Gamma oscillations play an important role in cognitive processes, including emotional processing in humans. Abnormal gamma oscillations may reflect certain psychiatric disorders, such as major depressive disorder (MDD). However, less attention has been paid to the role of gamma oscillations in treatment-resistant depression (TRD) and their association with the response to repetitive transcranial magnetic stimulation (rTMS).

**Methods:**

A total of 61 TRD patients were recruited for a two-week rTMS treatment consisting of ten sessions. Clinical assessments and eyes-closed resting-state electroencephalogram (EEG) recordings were conducted before and after treatment. Participants were randomly assigned to one of three treatment groups: prolonged intermittent theta burst stimulation (piTBS), 10-Hz rTMS, or a Sham group. Adaptive nonlinear analysis using Holo–Hilbert spectral analysis (HHSA) was applied to extract nonlinear information from the EEG data.

**Results:**

Gamma oscillations were found to be positively correlated with scores on the Hamilton Depression Rating Scale (HDRS-17). Additionally, changes in alpha-beta amplitude modulation (AM) modulated gamma oscillations were significantly larger in the Sham group compared to the two active stimulation groups. Furthermore, alpha-beta AM modulated gamma activity was significantly lower in responders compared to non-responders prior to rTMS treatment, irrespective of the specific rTMS protocol.

**Conclusions:**

Gamma oscillations may serve as an electrophysiological marker for the severity of depression in TRD. Additionally, alpha-beta AM could represent a potential predictor of response to rTMS treatment, identifiable prior to the treatment.

**Clinical trials registry number:**

UMIN000020892.

## Introduction

Major depressive disorder (MDD) is increasingly recognized as a common mental illness. Currently, repetitive transcranial magnetic stimulation (rTMS) and its alternative protocol, intermittent theta burst stimulation (iTBS), have been validated for their antidepressant efficacy, especially in treatment-resistant depression (TRD) through several clinical studies, e.g., (Blumberger et al., 2018; Li et al., 2020). Despite this, the response rate to these treatments remained limited, with less than 50 % effectiveness (Li et al., 2014). This limitation has led to an increasing interest in identifying accurate electrophysiological biomarkers that might predict and improve treatment outcomes (Daskalakis, 2014). As part of this effort, a previous study has demonstrated the varied mechanisms of two stimulation methods in TRD by demonstrating distinct oscillatory dynamics (Tsai et al., 2022). Additionally, midline frontal theta activity has been shown to predict the treatment efficacy of 10-Hz rTMS (Li et al., 2016), but not to iTBS (Li et al., 2021), which implied that two methods were required to be investigated for the possible markers.

As various studies have shown that cross-frequency coupling (CFC) played a pivotal role in neural communications in several cognitive processes (Canolty & Knight, 2010; Canolty et al., 2006) and is involved in the disrupted dynamics in clinical populations, such as MDD (Yakubov et al., 2022). With regard to electrophysiological biomarkers in MDD, according to a review article by Tsai, Li and Juan (2023), numerous studies have investigated alpha and theta oscillations. Alpha oscillations were found to increase in prefrontal regions after rTMS treatment in responders (Noda et al., 2013), although some studies reported inconsistent results (Spronk et al., 2008). Additionally, theta oscillations may be a promising predictor of rTMS effects, as frontal theta at pretreatment stage (Li et al., 2016) and increased subgenual anterior cingulate cortex (sgACC) theta in responders (Narushima et al., 2010) have been linked to treatment outcomes. In contrast, gamma oscillations have received relatively less attention, despite growing interest in their role as a potential biomarker (Fitzgerald & Watson, 2018).

Additionally, most of these studies applied conventional analysis, such as fast Fourier transform (FFT) while the processes in MDD as well as the effect of brain stimulation in MDD were characterized by more complex and dynamic information which may not be clearly revealed by conventional analytical methods. To further examine the underlying dynamics and cross-frequency interactions, Holo–Hilbert spectral analysis (HHSA), which was first introduced by Huang et al. (2016), provided an advanced method for revealing nonlinear and non-stationary CFC patterns in brain activities. Although FFT remained widely used and powerful to show the frequency pattern of the brain waves, HHSA further captured the information of amplitude modulations (AM) and without relying on the assumptions of sinusoidal or stationary signals. In addition to measuring pairwise couplings, HHSA provided all potential modulating and carrier frequencies as well as produced the multiple dimensions’ representations such as AM, carrier frequencies and time. HHSA has been successfully applied in various cognitive and clinical domains, including visual perception (Juan et al., 2021; Nguyen et al., 2019), working memory (Chang et al., 2023), Alzheimer’s disease (Chu et al., 2023), and Parkinson’s disease (Chang et al., 2022). Building on this basis, the present study used HHSA to examine the dynamic characteristics of gamma oscillations in TRD and their potential relationship with treatment response.

Gamma oscillations (30–100 Hz) play a crucial role in various cognitive processes, including perception, attention, memory, and emotional processing (Başar, 2013; Yang et al., 2020). The oscillations have been extensively studied in relation to neuropsychiatric disorders, offering insights into how abnormal gamma activity may contribute to cognitive and emotional dysfunctions. Herrmann and Demiralp (2005) highlighted that gamma activity varies significantly across conditions such as reduced in Alzheimer's disease and negative symptoms of schizophrenia, while increased in positive symptoms of schizophrenia, ADHD, and epilepsy. These findings provided a broad understanding of the role of gamma oscillations in brain function and dysfunction. In the context of MDD, Fitzgerald and Watson (2018) suggested that gamma oscillations have emerged as a putative biomarker. Current evidence indicated that gamma power (Strelets, Garakh & Novototskii-Vlasov, 2007) and complexity (Akar et al., 2015) were larger in MDD compared to healthy individuals during resting state electroencephalogram (EEG). Moreover, Scangos et al. (2021) used amygdala gamma power as the index for delivering deep brain stimulation in an MDD patient, based on findings of a positive correlation between amygdala gamma power and the severity of depressed symptoms. While these findings implied that a reduction in gamma power during the resting state in MDD might be related to the efficacy of rTMS treatment, it is important to note that some studies, such as those by Noda et al. (2017) and Pathak et al. (2016), have reported a positive correlation between increased gamma power and symptom improvement, which offers an alternative perspective to our hypothesis. These findings highlighted the complexity of gamma oscillations’ role in MDD and suggested that their involvement in TRD and the effects of various rTMS treatments required further investigation.

In light of previous reports, increased gamma power may be a characteristic of MDD and was potentially correlated with symptom severity. This study aimed to confirm the positive relationship between gamma oscillations and TRD. If confirmed, gamma power was expected to be modulated by rTMS, serving as a potential index of antidepressant efficacy. Additionally, the study sought to investigate whether gamma power would be correlated with the improvement of symptoms and whether it could be a predictor of responder or non-responder at pretreatment stage. To address this, the present study built upon a previously published dataset (Tsai et al., 2022), which examined low-frequency oscillatory dynamics based on the neural entrainment hypothesis, which stated that the frequency of exogenous stimulation would entrain and cause neural oscillations to align with it. The study used HHSA on the resting EEG before and after different rTMS groups and discovered that theta-alpha AM was entrained in association with prolonged iTBS, while 10-Hz rTMS increased alpha oscillations. However, the role of gamma oscillations in TRD and their modulation by rTMS remained unclear. This study extended on previous study by examining high-frequency gamma activity and its AM features. Therefore, the study primarily employed HHSA and also included FFT methods for comparison. In order to provide new insights into the role of high-frequency dynamics in TRD and the prediction of rTMS treatment response, this study also aimed to determine whether standard spectral information is adequate to characterize gamma activity in TRD or whether AM features can provide additional insights. Thus, this study hypothesized that gamma power and its AM characteristics may serve as neurophysiological markers of rTMS response in TRD, detectable using advanced spectral analysis methods.

## Methods and materials

The present study applied the same dataset from our previous publication (Tsai et al., 2022). Therefore, the procedure and all the parameters were the same as in Tsai et al. (2022). The brief descriptions were as follows.

### Participants

Total of 61 treatment-resistant depressed patients aged from 21 to 70 were included. Participants were diagnosed with MDD according to *DSM-IV* criteria (American Psychiatric Association, 1994) and had demonstrated inadequate response to at least one antidepressant medication. In addition, inclusion required a Clinical Global Impression-Severity (CGI-S) score of 4 or higher, and a score of 18 or greater on the 17-item Hamilton Depression Rating Scale (HDRS-17) (Hamilton, 1960). Individuals with a diagnosis of bipolar I or II disorder, a history of psychotic or personality disorders, neurological conditions, e.g., stroke or seizures, the presence of brain implants or cardiac pacemakers, or those who were pregnant were excluded from the study. Finally, regarding the assignment to treatment groups, 19 participants were randomly assigned to prolonged iTBS (piTBS) group, 20 participants were in 10-Hz rTMS, and 22 participants were in Sham group. The clinical study was conducted in accordance with the Declaration of Helsinki and was approved by the local ethics review committee. The registration number in the University Hospital Medical Information Network Clinical Trials Registry is UMIN000020892.

### Procedure

**Fig. 1:**
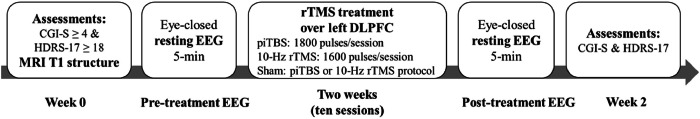
The experimental procedure. Participants underwent clinical assessments using the CGI-S scale and the HDRS-17, and completed a structural brain scan (T1-weighted MRI) at Week 0. Before the treatment phase, a 5-minute eye-closed resting-state EEG recording was collected. Participants were then randomly assigned to one of three rTMS treatment groups targeting the left dorsolateral prefrontal cortex (DLPFC), namely prolonged iTBS (1800 pulses/session), 10-Hz rTMS (1600 pulses/session), or Sham stimulation. Each participant received one session per day across five consecutive days per week for two weeks, totaling ten sessions. After completing the treatment, a second 5-minute eye-closed resting-state EEG was recorded. Finally, clinical assessments including CGI-S and HDRS-17 were repeated at Week 2.

Abbreviations: CGI-S, Clinical Global Impression-Severity scale; HDRS-17, 17-item Hamilton Depression Rating Scale; MRI, magnetic resonance imaging; piTBS, prolonged intermittent theta burst stimulation.

All TRD participants were first evaluated by using CGI-S and HDRS-17, and underwent T1-weighted structural MRI scanning to localize the stimulation site. Then their 5-minute eye-closed resting state EEG were recorded before (Week 0, W0) and after rTMS treatment (Week 2, W2). During the treatment phase, all participants were randomly assigned to one of three rTMS treatment groups (Group A: piTBS; Group B: 10-Hz rTMS; Group C: Sham). Each participant completed ten treatment sessions over a two-week period, receiving one session per day across five consecutive days each week. All participants were assessed using CGI and HDRS-17 following the completion of the treatment. The figure illustrating the procedure was reproduced from Tsai et al. (2022), Fig. 1, with permission (Fig. 1).

A double-blind design was used in this clinical trial, indicating that neither the doctors nor the participants recognized the treatment assignments. Each participant's assigned condition was only known to the staff members who were delivering the treatments. By minimizing participant expectancy effects and reducing evaluator bias during clinical assessments, this method improved the reliability of the results that were observed.

### Brain stimulation (piTBS/10-Hz rTMS) parameters

Magstim Rapid^2^ stimulator (Magstim Co., Ltd., Whitland, United Kingdom) was operated to deliver iTBS and rTMS with 70 mm figure-of-eight coil connected. Additionally, a sham coil was applied (Magstim Placebo Coil; Magstim Co., Ltd) to the Sham group which replicated the sound and tactile sensations of active stimulation without producing cortical activation. The parameters of iTBS and rTMS were in accordance with our previous study (Li et al., 2020). For the piTBS protocol, the study applied three consecutive sequences of the standard iTBS pattern, resulting in a total of 1800 pulses per session. The standard iTBS pattern consisted of 2-second bursts, repeated every 10 s, with each burst made up of triplets of pulses delivered at 50 Hz. These triplets were spaced every 200 milliseconds, corresponding to a 5 Hz rhythm. The stimulation intensity for piTBS was set at 80 % of the participant’s active motor threshold. For the 10-Hz rTMS protocol, stimulation consisted of 4-second trains of 10 Hz pulses, repeated every 30 s. The intensity was set at 100 % of the resting motor threshold, and each session delivered a total of 1600 pulses. For TRD in the Sham group, they were randomly assigned the protocols of either piTBS or rTMS. All the stimulation site was at the left dorsolateral prefrontal cortex (DLPFC), which was localized individually for each participant using MRI-guided neuronavigation. The localization was based on each participant’s T1-weighted MRI scan and was performed with the aid of a brain-navigation system and a Polaris infrared tracking device (Brainsight, Rogue Research, Inc., Montreal, QC, Canada).

### Electrophysiological recording parameters

A 36-channel which including two pairs of bipolar electrodes EEG cap (Quik-Cap) was used and connected to Neuroscan amplifier (NuAmps). The EEG signals were gained at 1000 Hz sampling rate without applying online filters. The average of electrodes at left and right mastoids (A1 and A2) was as the reference. Additionally, the electrooculograms (EOG) electrodes were placed around the eyes vertically and horizontally to detect eye movements. The impedances of all channels were maintained below 5 kΩ. All TRD participants have five-minute eye-closed resting EEG before and after the treatment.

### Data analyses

#### The determination of efficacy

The antidepressant efficacy was determined by the percentage changes in HDRS-17 score between Week 0 (W0) and Week 2 (W2). The TRD who had fifty percent or above reduction of HDRS-17 score at W2 were defined as responders. The improvement rate of the symptoms was calculated by the following formula –(W2 – W0)/W0 × 100 %. The negative sign is included to ensure that when the score in the second week (W2) is lower than the initial score (W0), the calculated improvement rate is positive. This adjustment simplifies interpretation, as a larger positive value directly corresponds to greater symptom improvement, making the results easier to visualize and understand.

#### Resting EEG analysis

##### Pre-processing

The continuous EEG data were segmented into 7-second epochs with a 2-second overlap to minimize data loss caused by edge effects during spectral analysis. A 100 Hz low-pass filter and a notch filter were applied to remove high-frequency noise and line noise commonly encountered in the testing environment. Baseline correction, using an interval from negative to positive infinity, was performed to adjust for signal drift. Independent Component Analysis (ICA) was employed on each dataset to eliminate the ocular and muscle artifacts. Trials with severe drift or artifacts exceeding ±200 μV were excluded. The data has been normalized by dividing the standard deviation. All preprocessing steps and subsequent statistical analyses were conducted using SPM12 for MEG/EEG (Wellcome Department of Cognitive Neurology, London, UK).

##### Holo–Hilbert spectral analysis (HHSA)

After preprocessing the EEG signals, HHSA (Huang et al., 2016; Juan et al., 2021) was performed using customized MATLAB (MathWorks) scripts. The HHSA comprised of two-layer empirical mode decomposition (EMD) (Huang et al., 1998). The EEG signals were initially decomposed into several intrinsic mode functions (IMFs) from high to low frequency, forming the first layer of IMFs. The second layer of IMFs was then obtained by applying empirical mode decomposition to the envelope of each first-layer IMF. Each IMF would then have three features: first, the data must have zero crossings; second, the difference between the number of maxima and minima must be less than or equal to one; third, the average of the upper and lower envelopes must be approximately zero. An improved approach to address the mode mixing problem and enhance the signals’ spectral separation, the complete ensemble empirical mode decomposition with adaptive noise (CEEMDAN) method was conducted in the analysis (Colominas, Schlotthauer & Torres, 2014; Jaiswal et al., 2023; Torres et al., 2011; Tsai & Liang, 2021). The instantaneous carrier frequencies and the amplitudes of the first-layer IMFs were extracted using Quadrature, while the instantaneous amplitude modulation frequencies were derived from the second-layer IMFs. This information was then mapped into a three-dimensional space to construct the Holo–Hilbert spectra (HHS), with dimensions of amplitude modulation frequency × carrier frequency × time. Since temporal resolution was not a main focus in this study, the spectral power was summed across the time dimension to create a two-dimensional HHS, where the y-axis represents amplitude modulation frequency and the x-axis represents carrier frequency. The data were log-transformed for subsequent statistical analysis.

##### Fast Fourier transform (FFT)

The preprocessed data were analyzed using FFT. The latency window was set to match the HHSA analysis, ranging from 2 to 5 s. The power spectrum was computed using a Hanning window with frequencies spanning from 0.5 Hz to 90 Hz in 0.2 Hz increments. A logarithmic (log10) transformation was applied to the power data for each condition (pre- and post-treatment) to normalize the distribution of power values and thereby facilitate adherence to the assumptions required by statistical analyses (Gasser, Bächer & Möcks, 1982). Subsequently, based on the frequency ranges of interest identified by HHSA, and in order to compare the results, the FFT frequency range of 47–90 Hz was averaged to focus on gamma-band activity. The overall sum of the AM frequency power within the specified carrier frequency range from HHS is approximately equal to the power in the FFT spectrum for the corresponding frequency range.

#### Statistical analyses

The demographic and clinical data were statistically analyzed using PASW Statistics, Version 18.0 (SPSS, Inc., Chicago, IL). Continuous data, such as assessment scores between groups, were compared using one-way ANOVA, whereas categorical variables, such as gender distribution and response rates, were examined using Pearson's chi-square test. The percentage change in HDRS-17 scores at Week 2 was determined for each group, as well as for comparisons between the piTBS, rTMS, and sham conditions. Multiple testing was controlled for by applying the Bonferroni correction to post hoc comparisons. A two-sided p-value of less than 0.05 was considered statistically significant. Additionally, adjusted significance level was *p* < 0.0167.

For EEG statistical analyses, multichannel HHS and FFT spectrum were tested using Pearson’s correlations. The level of statistical significance was set at *p* < 0.05 (two-sided) and multiple comparisons were corrected by the false discovery rate (FDR). The correlation was performed between log gamma power and HDRS scores at the pretreatment stage. Based on the topographic map from HHS, the power values from 14 consistent significant channels (F3, FZ, F4, FC3, FCZ, FC4, C3, CZ, C4, CP3, CPZ, CP4, PZ, and P4) across alpha and beta AM frequency bands were extracted and averaged to do the following examination. For FFT spectrum, the power values from the same channels in the same gamma frequency range (47–90 Hz) with HHS but without AM information were extracted and averaged to do the same examination with HHS. The contrast of EEG activities of post-treatment and pre-treatment across three groups (piTBS, 10-Hz rTMS and Sham) were calculated. In terms of normality, the Shapiro-Wilk test revealed that the contrast values did not follow a normal distribution. Consequently, outliers were identified and excluded using 1.5 × interquartile range (IQR) method, often referred to as Tukey’s boxplot criterion, which is widely recognized as a standardized approach for detecting extreme values in data (Tukey, 1977). Specifically, data points above the third quartile (Q3) × 1.5 IQR, or below the first quartile (Q1) × 1.5 IQR were removed. The IQR is defined as Q3-Q1. Therefore, the rest of 57 data from HHS (number of data in each group: piTBS=17, rTMS=19, sham=21) and 59 data from FFT spectrum (number of data in each group: piTBS=18, rTMS=19, sham=22) were then applied to the rest of statistical analysis (the flow diagrams were presented in Supplementary Figs. S1 & S2). One-way ANOVA was applied to compare the changes of gamma activities between three groups. Pearson correlation was tested between the differences of gamma activities and the percentage of the HDRS improvement. Finally, the independent *t*-test was employed to compare the gamma powers in responder and non-responder at the pretreatment stage.

## Results

### Demographic results

Baseline demographic and clinical characteristics across the piTBS, 10-Hz rTMS, and Sham groups were reported in our previous study (Tsai et al., 2022), where no significant baseline differences were observed. In the present study, although several outliers were excluded based on contrasts between pre- and post-treatment EEG data, which was identified through both HHS and FFT spectrum analyses, the demographic and clinical profiles remained consistently non-significant for age, gender, baseline CGI-S scores, and baseline HDRS-17 scores across groups (Table 1).

**Table 1:** Demographics and clinical assessments across treatment groups.

	piTBS	10-Hz rTMS	Sham
**number of participants**	19^a^	20	22
data from HHS	17	19	21
data from FFT spectrum	18	19	22
**Age, years**	48.7 ± 14.4^a^	49.1 ± 14.8	48.5 ± 12.9
data from HHS	48.1 ± 14.4	48.5 ± 14.9	47.7 ± 12.6
data from FFT spectrum	47.7 ± 14.1	48.2 ± 14.5	48.5 ± 12.9
**Female**	14 a	14	16
data from HHS	12	13	16
data from FFT spectrum	13	13	16
**CGI-S(BL)**	4.3 ± 0.6^a^	4.6 ± 0.8	4.4 ± 0.6
data from HHS	4.3 ± 0.6	4.6 ± 0.8	4.4 ± 0.6
data from FFT spectrum	4.3 ± 0.6	4.6 ± 0.8	4.4 ± 0.6
**HDRS-17(BL)**	22.5 ± 3.2^a^	22.6 ± 3.3	22.6 ± 2.6
data from HHS	22.3 ± 3.3	22.7 ± 3.4	22.5 ± 2.6
data from FFT spectrum	22.4 ± 3.2	22.6 ± 3.4	22.6 ± 2.6
**% change at W2**	−40.9 ± 0.07^b^^,^⁎⁎	−30.2 ± 0.06	−14.8 ± 0.03
data from HHS	−44.7 ± 0.07⁎⁎	−31.2 ± 0.07	−13.5 ± 0.03
data from FFT spectrum	−42.2 ± 0.07⁎⁎	−29.0 ± 0.06	−14.8 ± 0.03
**Responder( %, week2)**	8 (42 %)^a^^,^⁎⁎	6 (30 %)⁎⁎	0 (0 %)
data from HHS	8 (47 %)⁎⁎	6 (32 %)⁎⁎	0 (0 %)
data from FFT spectrum	8 (44 %)⁎⁎	5 (26 %)⁎⁎	0 (0 %)

Abbreviation: BL = baseline; CGI-*S* = the Clinical Global Impressions Scale-Severity of Illness; FFT = Fast Fourier Transform; HDRS-17 = 17-item Hamilton Depression Rating Scale; HHS = Holo Hilbert Spectra; piTBS = prolonged intermittent theta burst stimulation; rTMS = repetitive transcranial magnetic stimulation; *W* = week.

a Values are mean ± *SD, N* or *N %*.

b Values are mean ± *SE*.

⁎⁎ *p* < 0.01, compared to Sham group (Bonferroni corrected).

Regarding treatment outcomes, a significant group effect was observed for the percentage change in HDRS-17 scores at Week 2 (data from HHS: *F*(2, 54) = 8.17, *p* < 0.01; FFT dataset: *F*(2, 56) = 6.33, *p* < 0.01). Post hoc analyses indicated that the piTBS group showed a significantly greater reduction in depressive symptoms compared to the sham group across all datasets (*p* < 0.01). No significant difference was found between the piTBS and 10-Hz rTMS groups (data from HHS: *p* = 0.29; data from FFT: *p* = 0.32).

The response rate at Week 2 differed significantly across groups (data from HHS: χ²(2) = 11.99, *p* < 0.01; data from FFT: χ²(2) = 11.68, *p* < 0.01), with the piTBS group as well as the 10-Hz rTMS group demonstrating a significantly higher response rate compared to the Sham group. These patterns remained consistent after excluding outliers, further supporting the reliability of the treatment effects.

#### EEG results

**Fig. 2:**
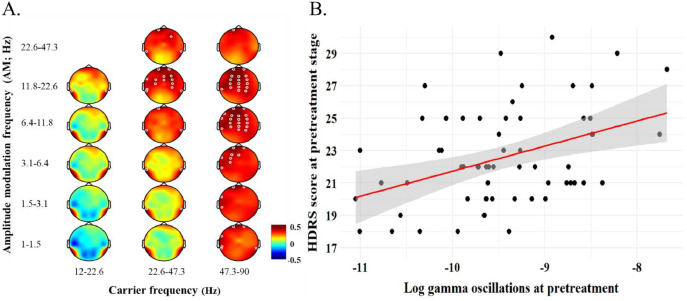
The correlation between resting EEG and HDRS scores in TRD at the pretreatment stage. A. showed positive correlation in the Holo–Hilbert spectra that x-axis indicated the carrier frequency and y-axis referred to the amplitude modulation (AM) frequency. The red color showed positive correlation while blue denotes negative correlation. The white circle in the figure showed the statistically significant channels. The false discovery rate (FDR) was applied for the multiple channels statistics correction. For emphasizing the most reliable effects, a stricter *p*-value threshold (*p* < 0.01) was applied when visualizing Holo–Hilbert spectral analysis (HHSA) results. B. demonstrated the scatter plot between gamma activities and HDRS score at pretreatment where the data was averaged from alpha-beta AM-modulated gamma bands (6.4–22.6 *f*_am_ | 47.3–90 *f*_c_) across the frontal, central, and parietal regions (F3, FZ, F4, FC3, FCZ, FC4, C3, CZ, C4, CP3, CPZ, CP4, PZ, and P4) based on the topography in Fig. 2A. The dark dots indicated each participants’ data points. Red line showed the regression line of the data and gray area referred to a 95 % confidence interval.

The relationship between baseline HDRS scores and HHS-based power of resting EEG at the pre-treatment stage was initially examined. Significant positive correlations were found in gamma bands, particularly in the beta AM-modulated low gamma (11.8–22.6 *f*_am_ | 22.6–47.3 *f*_c_) across widespread regions including frontal, central and parietal channels. Similar correlation patterns were found in the beta AM-modulated high gamma (11.8–22.6 *f*_am_ | 47.3–90 *f*_c_) and alpha AM-modulated high gamma (6.4–11.8 *f*_am_ | 47.3–90 *f*_c_), with significant effects covering frontocentral and posterior regions (see Table 2 for full significant channel list). The topographical plots for HHSA-based results in Fig. 2A displayed the channels with FDR-corrected *p*-values less than 0.01 to highlight the most reliable effects. Channels near the periphery of the electrode cap are more likely to be affected by noise. Therefore, the regions of interest were limited to 14 electrodes, including F3, FZ, F4, FC3, FCZ, FC4, C3, CZ, C4, CP3, CPZ, CP4, PZ, and P4, within alpha-beta AM-modulated high gamma bands (6.4–22.6 *f*_am_ | 47.3–90 *f*_c_). These channels were selected for further analysis based on their spatial consistency, statistical significance, and lower susceptibility to peripheral noise.

**Table 2:** Statistical results of HHS-based correlations between brain oscillations and HDRS scores at baseline.

Channel	*f*_am_: 6.4–11.8 Hz *f_c_*: 47.3–90Hz		*f*_am_: 11.8–22.6 Hz *f_c_*: 22.6–47.3Hz		*f*_am_: 11.8–22.6 Hz *f_c_*: 47.3–90Hz	
	r-value	FDR-corrected *p*-value	r-value	FDR-corrected *p*-value	r-value	FDR-corrected *p*-value
FP1	0.48	<0.001⁎⁎⁎	0.45	<0.001⁎⁎⁎	0.48	<0.001⁎⁎⁎
FP2	0.36	<0.01⁎⁎	0.36	<0.01⁎⁎	0.37	<0.01⁎⁎
F7	0.34	<0.01⁎⁎	0.32	<0.05*	0.33	<0.01⁎⁎
F3	0.40	<0.01⁎⁎	0.33	<0.01⁎⁎	0.41	<0.01⁎⁎
FZ	0.41	<0.01⁎⁎	0.35	<0.01⁎⁎	0.42	<0.01⁎⁎
F4	0.35	<0.01⁎⁎	0.42	<0.01⁎⁎	0.36	<0.01⁎⁎
F8	0.26	<0.05*	0.32	<0.05*	0.25	0.050
FT7	0.34	<0.01⁎⁎	0.34	<0.01⁎⁎	0.31	<0.05*
FC3	0.41	<0.01⁎⁎	0.36	<0.01⁎⁎	0.41	<0.01⁎⁎
FCZ	0.38	<0.01⁎⁎	0.38	<0.01⁎⁎	0.40	<0.01⁎⁎
FC4	0.37	<0.01⁎⁎	0.40	<0.01⁎⁎	0.37	<0.01⁎⁎
FT8	0.26	<0.05*	0.30	<0.05*	0.24	0.060
C3	0.37	<0.01⁎⁎	0.36	<0.01⁎⁎	0.38	<0.01⁎⁎
CZ	0.35	<0.01⁎⁎	0.26	<0.05*	0.37	<0.01⁎⁎
C4	0.34	<0.01⁎⁎	0.36	<0.01⁎⁎	0.35	<0.01⁎⁎
TP7	0.37	<0.01⁎⁎	0.34	<0.01⁎⁎	0.36	<0.01⁎⁎
CP3	0.36	<0.01⁎⁎	0.27	<0.05*	0.38	<0.01⁎⁎
CPZ	0.35	<0.01⁎⁎	0.29	<0.05*	0.35	<0.01⁎⁎
CP4	0.36	<0.01⁎⁎	0.35	<0.01⁎⁎	0.37	<0.01⁎⁎
TP8	0.29	<0.05*	0.31	<0.05*	0.28	<0.05*
T5	0.35	<0.01⁎⁎	0.23	0.081	0.36	<0.01⁎⁎
P3	0.32	<0.05*	0.22	0.089	0.33	<0.01⁎⁎
PZ	0.34	<0.01⁎⁎	0.22	0.082	0.34	<0.01⁎⁎
P4	0.36	<0.01⁎⁎	0.27	<0.05*	0.38	<0.01⁎⁎
T6	0.38	<0.01⁎⁎	0.28	<0.05*	0.36	<0.01⁎⁎
OZ	0.27	<0.05*	0.14	0.266	0.26	0.039
O2	0.31	<0.05*	0.16	0.220	0.31	0.014

⁎ FDR-corrected *p* < 0.05.

⁎⁎ FDR-corrected *p* < 0.01.

For visualize of these regions of interest, the scatter plot of the averaged values for these regions was created to illustrate the trends observed in the same dataset used to identify the frequency range of interest. As such, these visualizations are not independent evidence of the effect but rather a representation of the data used for selection (Fig. 2B), *r(60)* = 0.40, *p* < 0.05. The frequency range was then applied to further examinations.

**Fig. 3:**
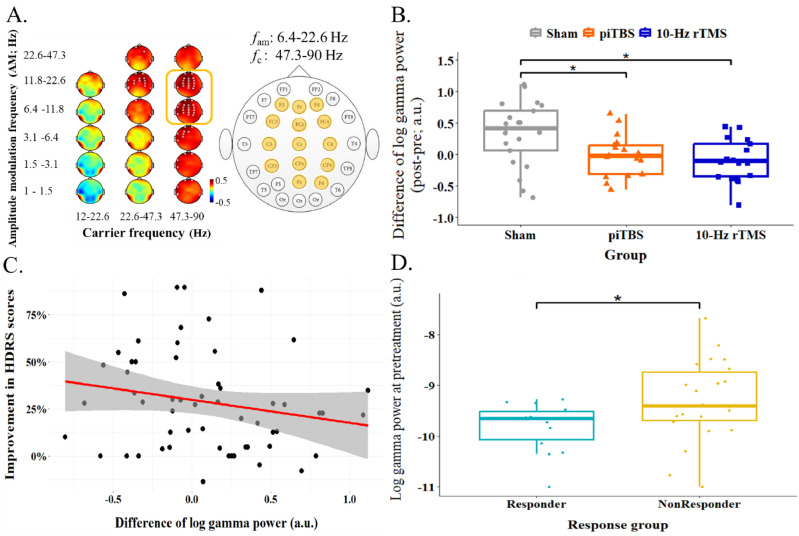
The examination of the gamma activities within the interest frequency ranges in the regions of interest from HHS. A. reiterated the aim frequency ranges over 6.4–22.6 *f*_am_ | 47.3–90 *f*_c_ and the regions of interest were marked in yellow color in the channel layout. The gamma power from these regions within the frequency ranges were averaged and thereafter for conducting following examinations. B. illustrated the treatment group differences of the changes in gamma activity by one-way ANOVA. The difference of gamma activity was increased in Sham and significant different from piTBS as well as 10-Hz rTMS. C. demonstrated a scatter plot to show the association between the changes of gamma power and improvement of the depressed symptoms. The results showed the trend of negative correlation whereas did not show significant statistically. Red line showed the regression line of the data and shadow area referred to a 95 % confidence interval. D. showed the independent *t*-test result of the comparison between the gamma activity at the pretreatment stage in responder and nonresdponder. The patients who have lower gamma activity before rTMS treatment was prone to be responder compared to non-responder.

Note. * p < 0.05.

Following the observed positive correlation of gamma oscillations and the scores in depression symptoms, gamma oscillations were hypothesized to be modulated by rTMS treatment. The changes of log gamma power in three groups, i.e., Sham, piTBS and 10-Hz rTMS in alpha-beta AM-modulated gamma frequency range was examined by one-way ANOVA. Differences of log gamma power were significantly larger in Sham group compared to piTBS and 10-Hz rTMS group, *F*(2, 54) = 6.23, *p* < 0.01 (Fig. 3B). However, the differences of log gamma power and the improvement of depressive symptoms were not significantly correlated, *r*(57) = −0.2, *p* = 0.13 (Fig. 3C). Nevertheless, this frequency range of log gamma power at the pretreatment stage could differentiate responder and non-responder (data in Sham group were excluded in this comparison), *t*(34) = −2.11, *p* < 0.05 (Fig. 3D). The difference between responder and non-responder were not shown when separated two protocols of rTMS, *F*(3,35) = 1.3, *p* = 0.29.

**Fig. 4:**
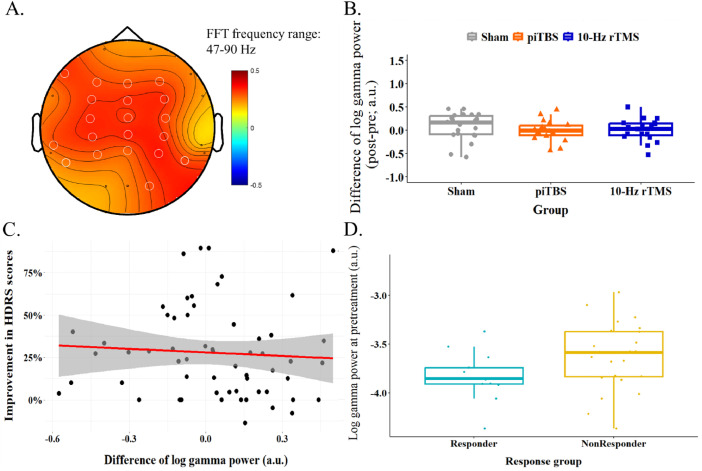
Fast Fourier analysis results included the examination of the gamma activities within the interest frequency ranges (47–90 Hz) in the regions of interest. A. showed the topography of positive correlation between 47–90 Hz brain activity and HDRS scores before the treatment. White circles indicated the statistically significant channels (FDR-corrected *p* < 0.05). Most of the them were consistent with HHSA results. The gamma power from certain 14 channels were averaged and thereafter for conducting following examinations. B. showed no differences of changes in gamma power between three groups. C. demonstrated no correlation between the changes of gamma power and improvement of the depressed symptoms. D. revealed no different between the gamma activity at the pretreatment stage in responder and non-resdponder.

In order to exam whether the gamma activity without AM information could play the same role as findings above, FFT was conducted to the same preprocessed data. A significant positive correlation was also found between gamma activity (47–90 Hz) and HDRS scores before the treatment. The significant channels are F7, F3, FZ, F4, FC3, FCZ, FC4, C3, CZ, C4, TP7, CP3, CPZ, CP4, P7, P3, PZ, P4, P8, O2 (see Table 3). The topographical plots for FFT-based results in Fig. 4A displayed the channels with FDR-corrected *p*-values less than 0.05, as no channels met the stricter significance threshold (*p* < 0.01) applied in the HHSA results shown in Fig. 2A. The significant regions were large overlap with HHS results. Same channels with HHS results (F3, FZ, F4, FC3, FCZ, FC4, C3, CZ, C4, CP3, CPZ, CP4, PZ, and P4) were used to conduct the rest of analyses. The difference of log gamma power across three groups showed in Fig. 4B that no significant difference was found in one-way ANOVA result, *F*(2, 56) = 0.81, *p* = 0.45. No correlation was observed between the improvement of symptoms and difference of log gamma power, *r*(57) = −0.07, *p* = 0.62 (Fig. 4C). Furthermore, gamma power was no significant different between responder and non-responder at the pretreatment stage, *t*(35) = −1.92, *p* = 0.06 (Fig. 4D).

**Table 3:** Statistical results of FFT-based correlations between brain oscillations and HDRS scores at baseline.

Channel	*f_c_*: 47–90Hz	
	r-value	FDR-corrected *p*-value
F7	0.32	<0.05*
F3	0.31	<0.05*
FZ	0.30	<0.05*
F4	0.30	<0.05*
FC3	0.33	<0.05*
FCZ	0.33	<0.05*
FC4	0.33	<0.05*
C3	0.34	<0.05*
CZ	0.32	<0.05*
C4	0.31	<0.05*
TP7	0.29	<0.05*
CP3	0.33	<0.05*
CPZ	0.34	<0.05*
CP4	0.33	<0.05*
P7	0.29	<0.05*
P3	0.29	<0.05*
PZ	0.28	<0.05*
P4	0.35	<0.05*
P8	0.33	<0.05*
O2	0.30	<0.05*

⁎ FDR-corrected *p* < 0.05.

In sum, gamma activities that specific to the alpha-beta AM-modulated gamma frequency (6.4–22.6 *f*_am_ | 47.3–90 *f*_c_) may serve as a putative electrophysiological marker for rTMS response prediction.

## Discussion

The demographic characteristics in the current study are generally consistent with those reported in Tsai et al. (2022), although some participants were excluded because of methodological requirements unique to each analytical approach such as HHSA and FFT. This supported the validity of cross-method comparisons by indicating that the analytic subsamples are demographically balanced. As a result, there are less concerns about sample bias or baseline group differences because the comparisons between measures derived from the HHSA and FFT are based on theoretically equivalent participant profiles.

The role of gamma oscillations in depression has recently received more attention whereas lacking of attention on rTMS response in treatment resistant depression. The present study investigated the relation between gamma oscillations and the depressed symptoms prior to the rTMS treatment was positively correlated. The results may imply that higher gamma activities at the eyes-closed resting state in TRD could serve as a marker represent the severity of the depressed symptoms. Furthermore, the gamma activities, especially alpha-beta AM modulated gamma oscillations, could be modulated by TMS treatment, though not specific to any rTMS protocols. The effect of neuromodulations make the level of gamma activity stable which was observed by the comparisons with the Sham group that gamma activities enhanced significantly during the treatment period without brain stimulations. However, the changes of gamma activities exhibited the trend of negative correlation with the improvement of the symptoms but did not reach statistical significance. These results implied that the changes of gamma activities may play a minor role in the treatment efficacy, although the small sample size could have limited the power to detect a significant effect. Nevertheless, the gamma power especially in the alpha-beta AM modulated gamma frequency range before treatment could differentiate responder and non-responder which may serve as a predictor of response to rTMS treatment.

Previous studies provided abundant evidence supports the cortical inhibition deficit hypothesis of MDD, which may give rise to the dysregulation of emotion and cognition (for a review see Croarkin, Levinson & Daskalakis, 2011). The cortical inhibition function in human play an important role to reduce the aberrant neuronal firing which mainly derived from the GABAergic system. The neurophysiological evidence showed impaired short-interval cortical inhibition (SICI), long-interval cortical inhibition (LICI) and cortical silent period (CSP) in MDD implied the impairment of cortical inhibition in MDD, which could be originated from the dysfunction of GABAergic system (Bajbouj et al., 2006; Jeng et al., 2020; Levinson et al., 2010). Ranging from molecular to neuroimaging evidence, including Magnetic Resonance Spectroscopy (MRS) studies, GABAergic interneurons in terms of both size and density and GABA level were lower in MDD contrasted to the comparison groups (Gerner & Hare, 1981; Gold et al., 1980; Hasler et al., 2007; Maciag et al., 2010; Price et al., 2009; Rajkowska et al., 2007; Sanacora et al., 1999). Furthermore, several studies showed the increment of GABA level after the pharmacological therapy (Sanacora et al., 2002) or rTMS treatment (Dubin et al., 2016; Levitt et al., 2019) in MDD by MRS studies as well as the increment of CSP after taking selective serotonin reuptake inhibitors (SSRIs) (Robol, Fiaschi & Manganotti, 2004) which referred to the treatment efficacy result in the improvement of cortical inhibitory function.

In regard to the relation between gamma oscillations and GABAergic system, few studies showed the relationship between them. Farzan et al. (2009) have applied a paired-pulse TMS paradigm, LICI that reflected GABA_B_ receptor mediated inhibition, and accompanied EEG recording to investigate the cortical inhibition in DLPFC in healthy human being. The results showed that gamma oscillations were significantly suppressed by LICI in the DLPFC, which suggested the critical role of gamma oscillation in cortical inhibition. One study in the rat that observed the EEG activation in its hippocampus by applying GABA_B_ receptor blockade. The results showed the increment in theta and gamma activations in the walking rat (Leung & Shen, 2007). In another recent animal study, the freely moving rat was injected by diazepam, which is the GABA_A_ receptor modulator that increased the efficiency of GABA binding to GABA_A_ receptors, while open-field EEG was simultaneously recording. The results showed that EEG features were associated with excitatory-inhibitory balance modulation. Specifically, high gamma oscillations (60–150 Hz) was suppressed compared to vehicle, which was the control condition in the frontal region illustrated in the power spectrum (Gonzalez-Burgos et al., 2023). These lines of evidence demonstrated certain indirect negative correlation between gamma oscillations and inhibitory function. Therefore, the hyper-gamma activities corresponding to the severity of depression in the present study echo previous studies and this index may reflect impaired cortical inhibition mechanisms in TRD.

Regarding the rTMS effects in the present study, gamma power remained stable following piTBS and 10-Hz rTMS, whereas the Sham group exhibited a significant increase in gamma power. This imply that piTBS and 10-Hz rTMS may influence GABAergic neurons. Although gamma power did not decrease following rTMS, the observed power increase in the Sham group, which occurred without brain stimulation, highlights the potential modulatory effects of rTMS. Moreover, rTMS was found to enhance inhibitory processes, further supporting its influence on neural activity. For instances, Daskalakis et al. (2006) applied high frequency (10 Hz and 20 Hz) rTMS on the motor cortex of healthy subjects and found the longer cortical silent period (CSP) after stimulations. Harrington and Hammond-Tooke (2015) investigated the increment of N100 amplitude of TMS-evoked potential, which might possibly indicate the enhanced GABA_B_ receptor activity, after applying iTBS in healthy individuals. Nevertheless, these were the late inhibitory processes or outcome, the underlying complex molecular mechanisms of iTBS and rTMS on cortical inhibition might be associated with the excitatory and inhibitory balance that involving glutamatergic and GABAergic systems which warranted further investigations (Li et al., 2022, 2019). As a result, the changes of gamma oscillations play minor role in the efficacy of rTMS treatment may be reasonable.

There could be some nonspecific explanations for the increase in gamma oscillations observed in the Sham group. First, spontaneous fluctuations in gamma oscillation could contribute to changes in gamma power over time. Resting-state gamma power is known to be inherently dynamic and sensitive to internal emotional and cognitive processes, even in the absence of external interventions. This may be a reflection of natural dynamic changes in mood states, vigilance, or emotional regulation over time (Başar, 2013). Furthermore, these dynamic changes may be even more varied in people with mental illnesses, such as depression (Fitzgerald & Watson, 2018) and schizophrenia (Uhlhaas & Singer, 2010), which might reflect their variable activities of the inner state. Second, the placebo or expectancy effects may have modulated neural activity which may be indirectly contribute to alterations in gamma oscillatory power. Previous study showed that the placebo treatments can activate specific brain pathways and neurotransmitter systems, such as endogenous opioids and dopamine, leading to measurable physiological and clinical changes (Benedetti et al., 2005). Additionally, many studies showed that brain oscillations such as gamma oscillations may be altered by neurochemical modulation. For instance, the neurotransmitters such as dopamine and acetylcholine are important in determining gamma activity association with perception, attention, and cognitive integration (Herrmann, Munk & Engel, 2004). Lastly, a greater degree of the severity of the symptoms may be the contributing factor to the increase in gamma power observed in the sham group following the two-week period. Unexpectedly, there was not a correlation between the severity of TRD in the Sham group and the increase in gamma power. This interpretation remains theoretical due to several limitations, including the short observation period, the small sample size, and the limited variation in symptom severity. The participants in the Sham group were already experiencing severe depression, as evidenced by their baseline HDRS-17 scores exceeding 18, which may have constrained the detectable range of symptom change. To confirm this possible association, larger and more diverse sample sizes will be required in future research.

In light of the prediction of rTMS treatment efficacy, alpha-beta AM modulated gamma appeared to play a more critical role than overall gamma oscillations. This may underscore the significance of CFC in brain functions (Canolty & Knight, 2010). This finding aligned with numerous previous studies emphasizing the importance of CFC in various cognitive processes that required interactions between neuronal oscillations at different frequencies. For example, Nguyen et al. (2019) revealed that low-frequency AM played a critical role in the dynamics processes of visual system. Additionally, Liang et al. (2021) demonstrated the importance of AM within frontoparietal beta oscillations and its inter-areal coupling with theta-band activity in visual working memory maintenance, highlighting how AM strengthens communication between brain areas. Furthermore, Chang et al. (2023) investigated parieto-occipital alpha/beta AM can predict working memory precision, indicating that AM play a central role of in facilitating communication with frontal theta oscillations to support working memory performance.

The present study showed that lower alpha-beta AM modulated gamma oscillations at baseline were more likely to predict responsiveness to rTMS treatment. This finding aligned with the perspective of Wagner et al. (2019), who mentioned that alpha-gamma coupling may reflect cortical excitability states and regulate neural processing windows where alpha oscillations may gate irrelevant information, allowing gamma bursts to process relevant stimuli. When this CFC was abnormally synchronized with excessive couplings, the regulation of cortical excitability and plasticity might influence the network interaction and cognitive function such as emotional dysregulation. In our results, reduced alpha-gamma coupling prior to the rTMS may provide greater modulating space, allowing rTMS to enhance cortical coordination and improve interactions, making individuals more likely to respond to treatment. In contrast, overall gamma oscillations combined information across all AM frequencies, which may potentially obscure the role of individual coupling mechanisms in predicting treatment outcomes. However, the physiological mechanisms of amplitude modulation required additional studies for clarification and validation. In addition to differences in mean gamma power, non-responders exhibited greater variability in gamma oscillations at baseline (responder: −9.81±0.49; non-responder: −9.25±0.82). Instead of consistently high gamma power, this pattern might be the result of inefficient excitatory–inhibitory balance or unstable local neural activity. This kind of variation might be an indication of abnormal cortical dynamics and impair the communication with and reaction to brain stimulation. This interpretation may be supported by the study from Jaworska et al. (2018), who demonstrated that non-responders showed higher EEG signal variability at fine temporal scales, especially in frontocentral regions, by using multiscale entropy (MSE). The authors indicated that this variability was a result of reduced global integration and excessive and dysregulated local processing. Despite the fact that the present study employed power-based analysis instead of multiscale entropy, both methods could be used to measure impaired cortical coordination and underlying network dysfunction. These findings indicated that dynamic EEG characteristics should be further investigated as potential biomarkers for rTMS response in TRD and implied that gamma activity variability may offer additional predictive value beyond average power measurements.

Because the number of responders in one rTMS treatment condition was limited, therefore, the findings of alpha-beta AM modulated gamma oscillations prior to the rTMS treatment, which could differentiate responders and non-responders, required further large-scale, sham-controlled studies to confirm the predicting effects in each rTMS protocol. In addition, the emerging biomarker of gamma oscillations require comparisons with healthy controls to validate the actual gamma power values at the pretreatment state for a reliable prediction. Currently, the marker is still not applicable for clinical practice due to the high variability caused by individual differences. However, larger sample sizes may enhance the predictive sensitivity and accuracy in the future.

## Conclusion

The study demonstrated the significant role of gamma oscillations in the severity of TRD. Furthermore, alpha-beta amplitude-modulated gamma oscillations, identified through adaptive nonlinear analysis using HHSA, were found to be a potential predictor of response to rTMS treatment. These findings provide important implications for the future development of personalized neuromodulation strategies, enabling the identification of individuals most likely to benefit from rTMS. Additionally, the study highlights the need for further research to validate these biomarkers and explore their physiological significance in larger, well-controlled clinical trials.

## Declaration of generative AI and AI-assisted technologies in the writing process

During the preparation of this work, the authors used ChatGPT to refine and improve the readability of the manuscript. After utilizing this tool, the authors thoroughly reviewed and edited the content as needed and take full responsibility for the final published article.

## Author contributions

C.H.J., C.T.L., designed the experiments. Y.C.T., C.M.C., J.S.J. and C.T.L. collected the data. Y.C.T. and W.K.L. analyzed the data. All authors reviewed the manuscript.

## Declaration of competing interest

The authors declare that they have no known competing financial interests or personal relationships that could have appeared to influence the work reported in this paper.

## Data Availability

The data that support the findings of this study are available from the corresponding author upon reasonable request.

## Appendix Supplementary materials

Image, application 1

Image, application 2
