# Supplementary material for: Amplitude modulated gamma oscillations as electrophysiological markers for repetitive transcranial magnetic stimulation efficacy in treatment-resistant depression: a randomized sham-controlled study
Source: Int J Clin Health Psychol. 2025 Jun 16;25(3):100593. doi: 10.1016/j.ijchp.2025.100593 (PMC12213092; doi:10.1016/j.ijchp.2025.100593)
Supplement: Supplementary file 1 [file mmc1.docx]

**Supplementary**

| **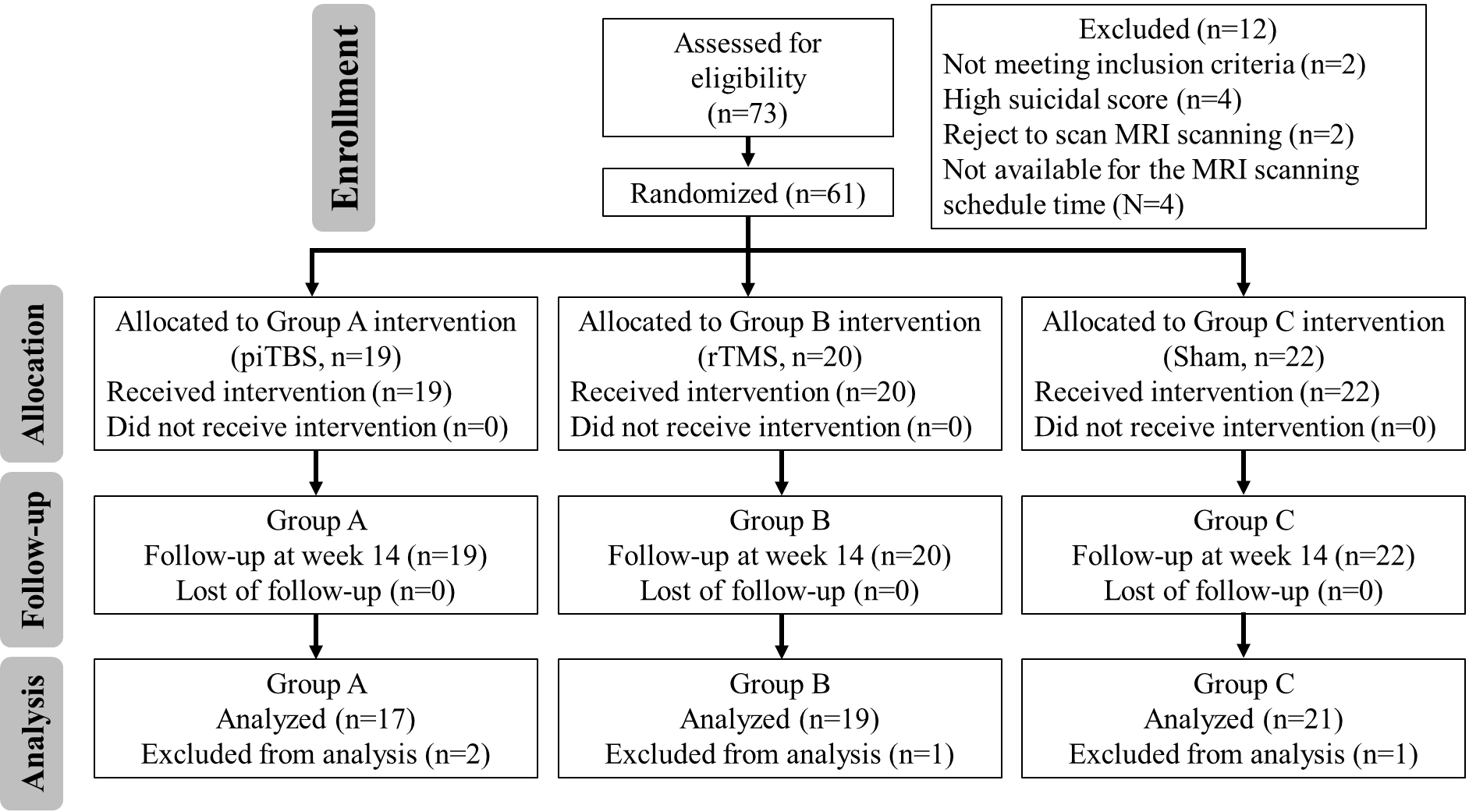** |
| --- |
| Figure S1. The flow diagram for Holo-Hilbert spectral analysis. |

| **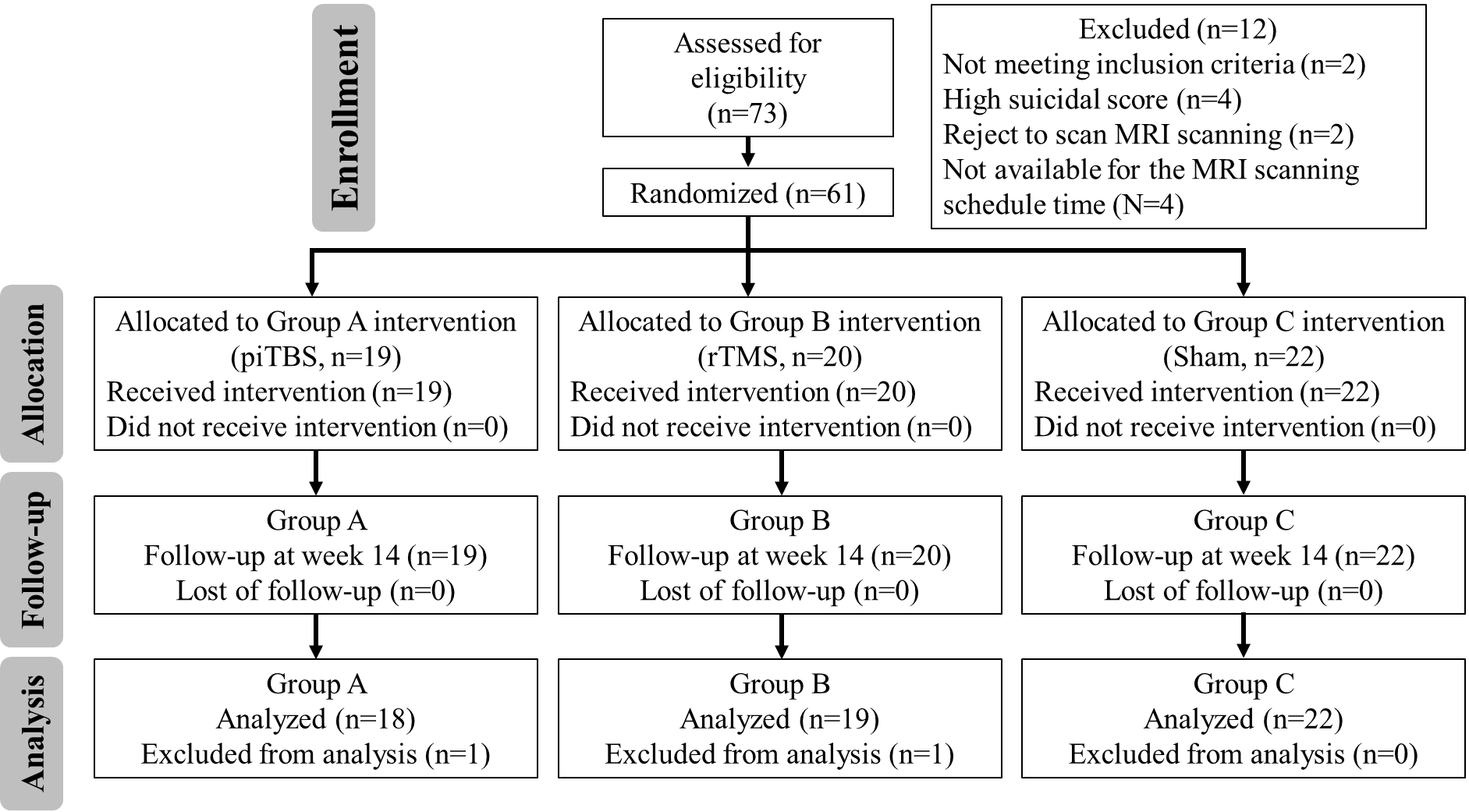** |
| --- |
| Figure S2. The flow diagram for Fast Fourier transform analysis. |
